# Effectiveness of the Unified Barlow Protocol (UP) and neuropsychological treatment in cancer survivors for cognitive impairments: study protocol for a randomized controlled trial

**DOI:** 10.1186/s13063-022-06731-w

**Published:** 2022-09-30

**Authors:** Francisco García-Torres, Adrián Tejero-Perea, Ángel Gómez-Solís, Rosario Castillo-Mayén, Maria José Jaén-Moreno, Bárbara Luque, Mario Gálvez-Lara, Araceli Sánchez-Raya, Marcin Jablonski, Beatriz Rodríguez-Alonso, Enrique Aranda

**Affiliations:** 1 Department of Psychology, University of Cordoba, Cordoba, Spain; 2 Maimonides Biomedical Research Institute of Cordoba (IMIBIC), Cordoba, Spain; 3 Reina Sofía University Hospital of Cordoba, Cordoba, Spain; 4 Department of Social Health Sciences, Radiology and Physical Medicine, University of Córdoba, Córdoba, Spain; 5 Collegium Medicum Jan Kochanowski, University in Kielce, Kielce, Poland; 6 Medical Oncology Department, Reina Sofía University Hospital, Córdoba, Spain

**Keywords:** Oncology, Cancer, Unified Protocol for Transdiagnostic Treatment of Emotional Disorders (UP), Cognitive impairment, Quality of life, Anxiety and depression

## Abstract

**Background:**

Cancer survivors frequently develop cognitive impairment, which negatively affects their quality of life and emotional well-being. This study compares the effectiveness of a well-established treatment (neuropsychological treatment) with the Unified Protocol for Transdiagnostic Treatment of Emotional Disorders (UP) to reduce these cognitive deficits and evaluate the effect of both treatments on anxiety-depressive symptoms and the quality of life of cancer survivors.

**Methods:**

A three-arm, randomized superiority clinical trial with a pre-post and repeated follow-up measures intergroup design using a 1:1:1 allocation ratio will be performed. One hundred and twenty-three cancer survivors with mild to moderate cognitive impairment will be randomly assigned to one of the study interventions: a cognitive rehabilitation intervention group, an intervention group with UP intervention, or a control group on the waiting list. The primary outcome is to observe a significant improvement in cognitive function in both intervention groups and a significant decrease in emotional impairments in comparison with the waitlist group. Improvements in anxiety, depression, and quality of life are also expected as secondary outcomes. These results will be maintained at 6 months of follow-up.

**Discussion:**

The aim of this trial is to test the efficacy of the UP intervention in reducing cognitive deficits in breast cancer survivors. The results of this trial may be useful in reducing the presence of cognitive problems in cancer survivors and improving their emotional state and quality of life.

**Trial registration:**

ClinicalTrials.gov NCT05289258. Registered 12 March 2022, v01.

**Supplementary Information:**

The online version contains supplementary material available at 10.1186/s13063-022-06731-w.

## Administrative information

Note: the numbers in curly brackets in this protocol refer to SPIRIT checklist item numbers. The order of the items has been modified to group similar items (see http://www.equator-network.org/reporting-guidelines/spirit-2013-statement-defining-standard-protocol-items-for-clinical-trials/).

**Table:** 

Title {1}	Effectiveness of the Unified Barlow Protocol (UP) and neuropsychological treatment in cancer survivors for cognitive impairments: study protocol for a randomized controlled trial
Trial registration {2a and 2b}.	ClinicalTrials.gov ID: NCT05289258, Registered 12 March 2022, v01.
Protocol version {3}	v.01
Funding {4}	This research have received funding from: The European Regional Development Fund (ERDF) Operational Programme Andalusia 2014-2020, Grant number: 1380800-R.
Author details {5a}	García-Torres, Francisco^1,2^; Tejero-Perea, Adrián^1, 2, 3^; Gómez-Solís, Ángel^3^, Castillo-Mayén, Rosario^1,2^; Jaén-Moreno Maria José^2,4^; Luque-Salas, Bárbara^1,2^; Gálvez-Lara, Mario^1,2^; Sánchez-Raya, Araceli^1,2^; Jablonski, Marcin^5^; Rodríguez-Alonso, Beatriz^2,3^; Aranda, Enrique^6^. ^1^Department of Psychology, University of Cordoba, Cordoba (Spain) ^2^Maimonides Biomedical Research Institute of Cordoba (IMIBIC), Cordoba, Spain ^3^ Reina Sofía University Hospital of Cordoba, Cordoba, Spain ^4^Department of Social Health Sciences, Radiology and Physical Medicine, University of Córdoba, Córdoba (Spain). ^5^ Collegium Medicum Jan Kochanowski, University in Kielce, Kielce (Poland) ^6^ Medical Oncology Department, Reina Sofía University Hospital, Córdoba, Spain.
Name and contact information for the trial sponsor {5b}	Francisco García-Torres (Principal Investigator) Department of Psychology, University of Cordoba**.** Avda./San Alberto Magno S/N. P. C. 14071 Córdoba (SPAIN) E-mail: z12gatof@uco.es. Tlf: +34 957218847
Role of sponsor {5c}	The funding body will not intervene at any stage of the research (design; data collection; management; analysis; interpretation of the data; writing of the report and the decision to submit the report for publication).

## Introduction

### Background and rationale {6a}

Breast cancer remains a major health problem worldwide. In 2020, an estimated 2.3 million women were diagnosed with breast cancer and there were 685,000 breast cancer-related deaths globally. With a 5-year prevalence of 7.8 million cases, breast cancer is the most common cancer worldwide. Despite this, advances in treatment and prevention have led to breast cancer survival rates of up to 90%, especially in cancers diagnosed at an early stage [1]. In Spain, a similar pattern can be observed, with breast cancer being one of the most frequently diagnosed types of cancer. In 2020, there were 6651 cases of breast cancer-associated mortality in the country, and it is estimated that the incidence of breast cancer will be 34,750 cases in 2022 [2].

Alterations in cognitive functioning are frequently observed in patients who undergo chemotherapy as treatment for noncentral nervous system cancer. Previous systematic reviews have estimated that up to 75% of patients showed a cognitive decline after chemotherapy, particularly in the domains of memory, executive function, attention, concentration, and processing speed. These symptoms negatively impact on patients’ quality of life, including impairments in terms of autonomy, self-confidence, social relationships, and return to work or education [3–5]. These alterations in cognitive functioning are usually mild to moderate and frequently appear after chemotherapy. However, the persistence of these symptoms varies over time. For example, according to several studies, 15–35% of patients report experiencing changes in their cognitive functioning months after the end of treatment [3]. In breast cancer patients, these alterations are observed to appear after chemotherapy and continue to be present 1, 5, and even 20 years after treatment [4]. In this line, Wefel et al. [6] found that 71% of breast cancer patients showed cognitive impairment after chemotherapy, and 61% of patients showed cognitive impairment 6 months after completing treatment. These results are similar to those of Collins et al. [7], who observed cognitive impairment in 48% of breast cancer patients and found that more than a third continued to show this impairment 1 year after treatment. Despite these results, few studies have examined the long-term cognitive decline in patients, although evidence suggests an improvement in cognitive decline after 6 months of chemotherapy completion [5].

Several types of interventions, from pharmacological treatment to physical activity, have been developed to reduce cognitive impairment in cancer patients [3]. Among psychological interventions, cognitive behavioral therapy and cognitive training have shown positive results, although studies on these interventions have been carried out with limited samples of patients or without a control group [8–12]. However, since both anxiety and depression are related to cognitive impairment in cancer patients, a transdiagnostic intervention such as the Unified Protocol (UP) may be effective in reducing the presence of symptoms of cognitive impairment in these patients [13–16].

Therefore, the primary objective of this trial is to test the efficacy of the UP in reducing cognitive decline and improving the emotional well-being (anxiety and depression) and quality of life of patients. The secondary objective is to establish the efficacy of neuropsychological treatment to reduce cognitive impairment and improve the psychological well-being and quality of life in these patients

### Objectives {7}

The following research hypotheses have been formulated:

1. The UP will be more effective in reducing cognitive impairment, symptoms of anxiety, depression, and quality of life than the neuropsychological treatment at post-treatment and follow-up (6 months).
2. Patients in the UP and in the neuropsychological treatment group will obtain better scores in cognitive impairment and symptoms of anxiety, depression, and quality of life than those in the waitlist group at post-treatment and follow-up (6 months).

### Trial design {8}

**Fig. 1:**
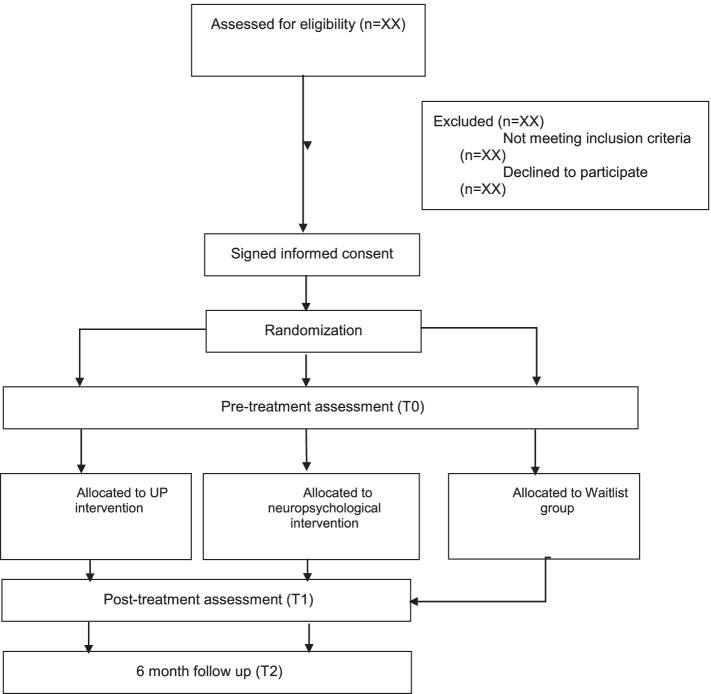
Study protocol flowchart

This is the protocol of a three-arm, controlled, randomized superiority trial, with a pre-post and repeated follow-up measures intergroup design. Participants with mild to moderate cognitive impairment will be randomly assigned to group 1: Unified Protocol for Transdiagnostic Treatment of Emotional Disorders (UP) intervention, group 2: Neuropsychological intervention, or group 3: Waitlist control group. For ethical reasons, the participants in group 3 will be offered the option to receive the neuropsychological intervention. The evaluation will be carried out at three time points: an initial evaluation prior to randomization (pre-treatment; T0), at the end of the intervention (post-treatment; T1), and at 6 months after completing the intervention (T2) (see Fig. 1).

## Methods: Participants, interventions, and outcomes

### Study setting {9}

This clinical trial will be conducted at the Reina Sofía University Hospital of Cordoba, Spain, and at the Faculty of Medicine of the University of Cordoba, Spain. The Reina Sofía University Hospital provides patients integral care, including diagnosis, cancer staging, specific oncological treatment, continuous care, palliative care, and disease follow-up care. The research group is responsible for the study design, the training of the personnel involved in the data collection, the application of the interventions, the provision and preparation of all study documents, the supervision of the study, the treatment and analysis of the data obtained, and to communicate and disseminate the research results.

### Eligibility criteria {10}

Inclusion criteria in the trial: Men and women aged 18 to 70 years. Cancer diagnosis, stages I-III. Cancer type: Breast. The participants must have received the last chemotherapy session in the last 6 months and completed a maximum of 6 years of treatment. Probable or mild to moderate cognitive impairment (score of 10–26 points on the Mini-Mental State Exam [MMSE]). Fluent in Spanish. Not currently participating in another clinical trial. Not currently receiving other psychological treatment.

Exclusion criteria in the trial: Men and women aged > 70 years. Diagnosis of stage IV cancer or other types of cancer. Last chemotherapy session < 6 months or > 6 years. No cognitive impairment (MMSE score of 27–30 points). Diagnosis of mental disorder (including substance abuse) prior to cancer diagnosis. Disease relapse after completion of chemotherapy treatment. Diagnosis of neurodevelopmental disorder. Diagnosis of diseases that affect cognitive performance such as hypertension, cardiac diseases, epilepsy, dementias, multiple sclerosis, functional disorders (chronic fatigue syndrome, irritable bowel syndrome, post-concussion syndrome, whiplash syndrome), central nervous system (CNS) infections (HIV, encephalitis), metabolic disorders (diabetes, B12 deficiency), obstructive sleep apnea, brain damage (stroke, traumatic brain injury, CNS cancer). Use of medications/substances that interfere with cognitive function such as pregabalin, gabapentin, topiramate, antidepressants tricyclics, sodium valproate, anticholinergics, methylphenidate, or typical antipsychotics

### Who will take informed consent? {26a}

After recruitment, participants will be arranged to be screened for cognitive impairment using the MMSE and asked to sign the informed consent form. Participants will be provided with information sheets with general information about the study and will be offered the opportunity to ask any questions about their participation. If the participant agrees, he/she will be provided with a written informed consent that will guarantee the confidentiality of the data and the possibility to leave the study at any time without any adverse effects (revocation of informed consent). The informed consent will be collected by a member of the research team with training in clinical psychology.

### Additional consent provisions for collection and use of participant data and biological specimens {26b}

The data obtained will be used only for the purposes of this research and biological samples will not be collected.

## Interventions

### Explanation for the choice of comparators {6b}

This trial tests the efficacy of an adaptation of Barlow's Unified Protocol in reducing the presence of symptoms of cognitive impairment in cancer patients, compared to a cognitive rehabilitation program whose results have been shown to be effective in previous studies. This rehabilitation program includes training in memory and processing speed and has been tested in breast cancer patients treated with chemotherapy in randomized controlled trials, showing significant improvements in patients' cognitive functions after the intervention and at 2 months follow-up. The wait-list control group will be assigned to receive usual care [17–19].

### Intervention description {11a}

Unified Protocol for the Transdiagnostic Treatment of Emotional Disorders (UP). The UP intervention will focus on a deficit in emotional regulation common to all emotional disorders (ED). Specifically, the intervention will address the adaptive value of emotions and promote tolerance to intense emotions as well as identify and modify dysfunctional emotional regulation strategies. Patients will receive 8 group therapy sessions (8–10 cancer survivors per group) with all UP modules [20]. The Spanish version of the therapist’s guide and the patient’s workbook will be used [21]. All patients will receive the workbook to inform them of the contents of each session, aid them in doing the recommended exercises between sessions, and help them once the treatment is completed. The treatment will last 8 weeks (one session per week) as follows:

- *Session 1: Motivation for change and commitment to treatment.* The main objectives of the first session are to establish the therapeutic relationship and promote motivation for change. Therefore, tasks pursuing that goal will be carried out such as the analysis of the advantages and disadvantages of change and the setting of treatment goals or life goals.
- *Session 2*: *Understanding emotions.* The second session will focus on teaching the adaptive components of emotions in patients and will begin by working with the concept of emotion-driven behaviors (EDBs), differentiating between three emotional components: behaviors, thoughts, and physical reactions.
- *Session 3: Emotional awareness training. Learning how to observe experiences.* In this session, patients will be trained in emotional awareness through the practice of exercises focusing on the present without judging what they are perceiving. For example, exercises involving mood induction with music.
- *Session 4: Cognitive restructuring.* The objective of this session is to promote cognitive flexibility. To achieve this aim, participants will be trained to identify and modify non-adaptive thoughts (e.g., through cognitive restructuring or positive self-instruction).
- *Session 5: Emotional avoidance and EDBs.* This session will analyze emotional avoidance patterns and EDBs as well as their role in the development and maintenance of emotional disorders. In addition, patients will be trained to modify their dysfunctional behaviors.
- *Session 6: Physical sensations tolerance.* In this session, the objective is to train patients in improving their tolerance to physical sensations. To this end, participants will learn to identify uncomfortable inner physical sensations associated with emotions using breathing exercises.
- *Session 7: Situational and interoceptive emotional exposure.* The objective of this session is to expose the patient to emotions by promoting habituation instead of emotional avoidance. A situational and emotional avoidance hierarchy will be developed and followed by exposure exercises for that purpose.
- *Session 8: Maintenance of treatment gains and relapse prevention.* In the last session, participants will check their progress and what was learned in previous sessions will be recapitulated to promote the later use of this knowledge once the intervention is over. The aim is to provide patients skills to deal with demanding situations in the future.

Neuropsychological treatment consists of a combination of the programs tested by Von ah et al. [17]. These programs involve cognitive training adapted from the Advanced Cognitive Training for Independent and Vital Elderly (ACTIVE) intervention [18] and processing speed training using the InSight program (developed by Posit Science) [19].

The first memory training will consist of teaching the patients memorization techniques for remembering word lists, text material, and sequences. Participants learn how to apply principles of meaningfulness, clustering, organization, visualization, and association using strategies such as visual memory support, story mnemonic, and the method of loci [22]. These tasks will be performed in the first hour of every session. The sessions will be structured as follows: Sessions 1–4 will focus on strategy instruction and exercises to practice the strategy learnt in that session. In sessions 4–8 additional practice exercises to promote self-efficacy in terms of performance will be provided. The second program will consist of a series of exercises on processing speed of varying difficulty. Exercises include time-order judgment, discrimination, spatial-match, forward-span, instruction-following, and narrative-memory tasks. The InSight program automatically adjusts the difficulty of these exercises to users’ performance to maintain an 85% rate of correct performance [19]. In the same line, this program systematically reduces the stimulus duration during a series of progressively more difficult tasks that are displayed on a computer. These exercises will be performed in the second hour of each session throughout the entire intervention.

The intervention will be carried out in groups with a maximum of 5 participants per group [17] and consists of 8 weekly sessions of 2 h each: the first hour for memory training and the second for processing speed exercises (PSE). Contents for 8 weeks:

- *Session 1: Clustering strategies and PSE.* In the first session, the goal is to learn how to organize a list of verbal or visual stimuli into meaningful categories and clustering during the first hour. To achieve this goal, the psychologist will introduce the session and explain the strategies and exercises to practice them. In the second hour of the session, the participants will be trained in processing speed using the InSight program. The psychologist will remain in the room to answer any questions and support the participants while they do the computerized exercises.
- *Session 2*: *Summarizing strategies and PSE.* In the first hour of this session, participants will be trained to organize, summarize, and select the main ideas and details in order to remember text-based information. Once the strategies have been explained, paper-based exercises will be used. Later, patients will do processing speed exercises on computers under the psychologist’s supervision.
- *Session 3*: *Visualizing strategies and PSE.* The first hour of the third session focuses on teaching visualizing strategies to remember different items. In the same line as previous sessions, the strategy will be explained first and the practical exercises will be performed later. The second hour will focus on processing speed exercises using the Posit Science InSight program as well.
- *Session 4*: *Association strategies and PSE.* In the first hour of this session, the objective is to use association to remember items. In this case, the strategies will be taught using both visual and verbal materials. In the second hour, participants will be trained in processing speed as usual with the psychologist’s support when necessary.
- *Sessions 5, 6, 7, and 8: Revising strategies and PSE.* In the last four sessions, what was learned in the previous sessions will be entrenched with additional practice exercises in the first hour. Thus, mixed exercises will be done in these sessions where participants are asked to apply different strategies to each exercise and solve doubts. Likewise, processing speed training with the Insight program will be provided in the second hour.

Control group on waiting list. Once the interventions in groups 1 and 2 have been completed, the control group will receive the neuropsychological treatment described above. The group on the waiting list will participate in all the evaluations (T0, T1, and T2)

### Criteria for discontinuing or modifying allocated interventions {11b}

Participants who meet any of the following criteria over the course of the intervention will be excluded from the study: participants who request to withdraw from the study, fail to attend two or more appointments or suffer a relapse with a second cancer diagnosis

### Strategies to improve adherence to interventions {11c}

The therapist will emphasize the need to attend and complete the scheduled sessions of the proposed interventions and not to withdraw from them. To this end, emails and phone calls will be used during the intervention period to remind participants of appointments and tasks to be completed in order to improve their adherence to treatment.

### Relevant concomitant care permitted or prohibited during the trial {11d}

Participants who receive a second diagnosis of cancer or begin receiving another psychological intervention during participation in the trial will be excluded.

### Provisions for post-trial care {30}

No potential harm is expected in this trial.

### Outcomes {12}

#### Primary outcomes

Participants are expected to show an improvement in cognitive performance (FACT-COG, MFE-30, HVLT-R, TMT, and COWAT) following both interventions (UP and neuropsychological treatment) in the post-treatment (T1) with respect to the pre-treatment (T0). These positive changes are expected to remain stable for 6 months in follow-up (T2), but the participants in the UP intervention group are expected to obtain better scores than the cognitive neuropsychological intervention group. Therefore, better scores are expected in cognitive performance measures for the variables assigned to each test. Thus, no significant improvement in the waitlist control group is expected either at T1 compared to T0, or at T2 compared to T1. On the other hand, there is no broad statistical agreement about the cut-off for a clinically significant change in cognitive impairment [23]. In a longitudinal study, Hermelink et al. [23] considered that a change in cognitive impairment of 1 standard deviation (SD) from the norm indicates mild impairment and a change of 2 SD moderate impairment. Thus, the present study considers 1 SD and 2 SD from the mean in the score of each cognitive measure for T1 or T2 with respect to the initial measurement of each test (T0) as slight and moderate significant changes, respectively

#### Secondary outcomes

The secondary outcomes include changes in the level of QoL, anxiety, and depressive symptoms (QLQ C-30, BR23, and HADS) of the participants in both intervention groups, with better scores for the UP intervention group. Therefore, changes are expected in T1 with respect to T0 in the mean for QLQ C-30, BR23, and HADS scores and are expected to persist in T2. These scores are expected to remain stable in the waitlist group. The expected changes are manifested by a decrease in the score for each scale. Zwerenz et al. [24] used the HAD scale, specifically the depression subscale (HADD), to measure depressive symptoms and considered a variation of at least two points in the different measures as a clinically significant change in symptoms. Therefore, this study assumes that a variation of at least two points from the mean on the anxiety (HADA) and depression (HADD) subtests indicates a clinically significant change in symptom intensity. According to various authors, a change in QLQ C-30 scores of 5 to 10 can be considered as “little” change in terms of quality of life and of 10 to 20 as “moderate” change, while if this variation is greater than 20, the change is “very much” and a change of 10 points is considered meaningful [25, 26]. Thus, this study considers that a variation of 5–10 points, 10–20 points, and more than 20 points from the mean are considered as slight, moderate, and high significant changes, respectively.

### Participant timeline {13}

**Fig. 2:**
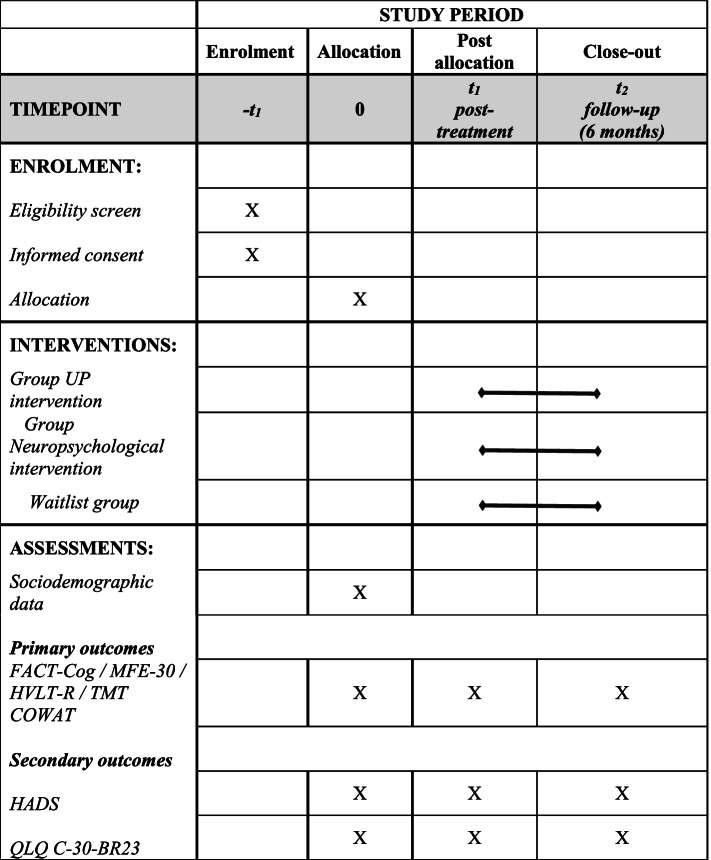
Schedule of enrolment, interventions, and assessments

The schedule for recruitment, interventions, assessment, and follow-up are shown in Fig. 2.

### Sample size {14}

The sample size has been estimated based on data obtained in a similar randomized trial with cancer patients of different types [12]. This study examined the effect of a cognitive rehabilitation program on cognitive impairment compared to a control group. The results showed a moderate effect size of 0.49 at post-treatment. Given these results, a medium effect size of 0.5 (*d* index) has been assumed to detect differences between the interventions and the waitlist group. Because no software was available to determine the sample size for linear mixed model analyses, we used the *f* index of G*Power. Therefore, an effect size of 0.25 (*f* index) is assumed, equivalent to *d* = 0.5. Thus, with a statistical power of 0.80, an alpha level of 0.05, a correlation between repeated measures of 0.5 and three groups, a total sample of 108 participants (36 participants in each group) is required. Previous studies have reported a participant withdrawal rate of around 13% [12]. Therefore, assuming this withdrawal rate, the total sample would be 123 participants, 41 per group.

### Recruitment {15}

The oncology team at the Reina Sofia University Hospital of Cordoba (Spain) will be responsible for assessing the suitability of the patients to enter into the study during their routine visits, based on their clinical histories and following the inclusion and exclusion criteria. Eligible participants will be contacted using a telephone by a member of the research team with formation in clinical psychology to provide an invitation to participate in the study. It is expected that a sufficient number of participants will be recruited to start the different interventions with the same number of participants at the same time.

## Assignment of interventions: allocation

### Sequence generation {16a}

Participants who obtain scores indicating mild or moderate cognitive deterioration will be randomly assigned to one of the three interventions (Neuropsychological treatment/Unified Protocol for the Transdiagnostic Treatment of Emotional Disorders (UP)/waitlist group) at a 1:1:1 ratio using a computer-generated set of numbers (https://www.randomizer.org/) and stratified by psychiatric medication and fibromyalgia diagnosis (if any).

### Concealment mechanism {16b}

The research team will receive information regarding the assignment of participants to the different intervention groups by means of sealed opaque envelopes

### Implementation {16c}

Randomization and allocation concealment will be conducted by independent researchers.

## Assignment of interventions: Blinding

### Who will be blinded {17a}

Participants will receive basic information about the study but not about the study objectives or which intervention is considered active. The psychotherapists involved in conducting the interventions will not receive detailed information about the study objectives. The members of the research team involved in assessing the participants will not receive information about the study objectives, the group assignment, or the results expected to be obtained in the study. In addition, the data analyst will be blinded to the allocation of participants to the different study groups.

## Procedure for unblinding if needed {17b}

Not applicable. Unblinding will not be carried out until the study is completed.

## Data collection and management

### Plans for assessment and collection of outcomes {18a}

#### Mini-Mental State Examination (MMSE)

The MMSE consists of a quantitative screening test of cognitive function comprising 11 questions that are administered between 5 and 10 min. Three items are scored from 0 to 5, 3 items are scored from 0 to 3, 1 item is scored from 0 to 2 and 4 items are scored from 0 to 1. The maximum score that can be obtained is 30, with a higher score indicating less cognitive impairment. A score below 24 points indicates possible cognitive impairment. The validity of MMSE has been demonstrated in a Spanish population with a Cronbach’s alpha of .94 [27, 28].

#### Functional Assessment of Cancer Therapy-Cognitive Function, version 3 (FACT-Cog)

This instrument was developed to assess chemotherapy-induced cognitive problems in cancer patients. This 37 items scale includes four different subscales: Perceived Cognitive Impairments, Impact of Perceived Cognitive Impairments on QoL, Comments from Others, and Perceived Cognitive Abilities. The sub-scales are scored using a 5-point Likert scale from 0 (*never*) to 4 (*several times a day*). Some items are reversed score. Higher scores reflect fewer cognitive problems and better QoL. This instrument has good psychometric properties with α = .94 [29, 30].

#### Memory Failures Everyday (MFE-30)

The MFE-30 is a unifactorial questionnaire that measures a single construct: “cognitive complaints.” It is made up of 30 items that are answered on a 5-point Likert scale from 1 (*never*) to 4 (*always or almost always*). The minimum score is 30 and the maximum score is 120, with higher scores indicating poorer memory function. The test has been validated in the general Spanish population with Cronbach’s alpha scores between .91 and .93 [31].

#### Hopkins Verbal Learning Test-Revised (HVLT-R)

This test measures primary and secondary memory, the rate of verbal learning throughout three trials, as well as three forms of mnesic organization: serial ordering, semantic grouping, and subjective organization. The test consists of a list of 12 words that are presented orally at a speed of one word for every 2 s. The person tested scores 1 point for every word remembered. The maximum total score after the three trials is 36 and the minimum score is 0. The more words remembered, the better the memory functioning. According to the International Cognition and Cancer Task Force (ICCTF) [32], the HVLT-R is one of the essential, recommended tests and has good internal consistency with *α* = .81 [33].

#### Trail Making Test (TMT)

The TMT consists of two parts: Part A measures attention, processing speed, visual search, and working memory, while Part B is used to measure attention, executive function (cognitive flexibility, ability to change tasks, coordination of categories), working memory, visual-motor skills, and processing speed. It uses time as a measure of cognitive performance. The less time it takes, the better the cognitive processing. Scores higher than 78 seconds on Part A and 273 seconds on Part B are considered deficient. The test shows good internal consistency (*α* = .83) [34]. Furthermore, the TMT is not language dependent, making it one of the three, highly recommended tests by the ICCTF [32].

#### Controlled Oral Word Association Test (COWAT)

The COWAT is a test that measures semantic and phonological verbal fluency and is a recognized and sensitive indicator of cognitive functioning. It aims to say as many words as possible during 1 minute in three trials. The higher number of said words indicates better verbal fluency. The internal consistency of the test is adequate (*α* = .86) [35, 36]. This is the third test recommended by the ICCTF for the assessment of cognitive impairments [32].

#### Hospital Anxiety and Depression Scale (HADS)

The HADS uses a 14-item scale with 7 items per subscale: HADA (Anxiety) and HADD (Depression). Items are scored from 0 to 3 with a total score for each scale of 0 to 21. The higher the score, the more pronounced the symptoms. The cut-off point for possible cases is more than 8 points [37]. The Spanish version of this scale has been validated in an oncological sample and shown an internal consistency of α = .85 for HADA and α = .87 for HADD [38].

#### EORTC QLQ C-30 (version 3) and BR23 breast cancer module

This instrument was developed to assess quality of life in cancer patients using 30 questions about the quality of life experienced by the patient during the past week. The first 28 items include questions about different symptoms and are answered on a scale of 1 (*not at all*) to 4 (*very much*), while the last two items ask patients to rate their self-perceived overall health and overall quality of life on a scale of 1 (*very poor*) to 7 (*excellent*). The items are grouped into functional scales (physical, role, emotional, cognitive, social, and global) and a symptom scale (fatigue, nausea and vomiting, dyspnea, sleep problems, loss of appetite, constipation, diarrhea, and financial impact). This instrument was developed to be administered to cancer patients and is widely used in studies with these patients. The Spanish version of the test has shown good psychometric properties (*α* > .70) [39]. The BR23 module adds 23 items rated from 1 (*not at all*) to 4 (*very much*) and it was developed to assess specific features in breast cancer patients using five, multi-item scales: body image, sexual functioning, systemic therapy side effects, breast symptoms, and arm symptoms. Additionally, single items assess sexual enjoyment, future perspectives, and being upset by hair loss. In EORTC QLQ C-30 (version 3) and BR23 breast cancer module, higher scores reflect a lower QoL in the different features assessed [40].

#### .Demographic Variables Questionnaire

This questionnaire was prepared by the researchers of this work to collect some sociodemographic and clinical variables that are intended to be analyzed such as age, sex, marital status, level of education, psychiatric medication (if any), type of cancer and cancer stage, kind of cancer treatment, time from cancer diagnosis to end of chemotherapy, and fibromyalgia diagnosis.

### Plans to promote participant retention and complete follow-up {18b}

In order to minimize loss to follow-up, once the intervention period is finished, participants who complete the intervention sessions will receive phone calls to remind them of their participation in the study and to set up the follow-up evaluation session.

### Data management {19}

The members of the research team in charge of collecting the data will receive prior training on the correct way to fill out the questionnaires, in order to unify criteria and resolve possible doubts before starting the evaluation. To ensure the accuracy of the data entry, it will be carried out independently by two researchers. If any discrepancies appear, they will be resolved by going back to the data source. Finally, the trial steering committee, consisting of the principal investigator and members of the research team, will be responsible for monitoring the quality and accuracy of the data.

To ensure participants’ anonymity, once they are included in the study, their information will be treated using a code number. Data obtained from the questionnaires and all sociodemographic and clinical data will be collected in paper format and stored in a locked filing cabinet during and after the trial. The researchers shall retain all data collected for up to 5 years after the end of the study.

### Confidentiality {27}

Participants must complete an MMSE screening test before entering the study. If the scores of the screening test indicate cognitive deterioration, the participant will be provided an informed consent with information about the trial, the confidentiality of the results, the possibility of withdrawing from the study at any time and without giving any reason, and no negative consequences in the event of withdrawal. All informed consent forms will be kept in a locked cabinet at the Reina Sofía University Hospital. The participants’ information will be encoded with a unique ID to ensure confidentiality and stored in a locked file at the hospital. The research team will only have access to the clean dataset with the encoded participant information. These datasets will be protected by a password and stored in a computer that will only be accessible to the members of the research team.

### Plans for collection, laboratory evaluation, and storage of biological specimens for genetic or molecular analysis in this trial/future use {33}

The data collected will be used only for the purposes of this research and no biological samples will be collected.

## Statistical methods

### Statistical methods for primary and secondary outcomes {20a}

The obtained data will be analyzed following the intention-to-treat (all randomized patients are included in the analysis) and per protocol (only patients with post-treatment assessment are included in the analyses) approaches. In an intention-to-treat analysis, incomplete or missing data will be considered using the maximum likelihood estimation method. First, ANOVA or chi-square tests will be performed to compare the demographic variables and outcome measures at baseline (T0). Second, to examine longitudinal changes over time (baseline, post-treatment, and follow-up) and between-group differences in the mentioned variables, mixed linear models will be used, since these models are more precise than repeated measures ANOVA [41]. The Friedman test will be performed to analyze a potential group-level effect among participants receiving the intervention together. In addition, Cohen’s *d* with bias corrections will be calculated to determine the effect size of the between-group comparisons. The most important analysis will be performed between the two active groups (the neuropsychological treatment group and the transdiagnostic treatment group) and the control group, as well as a comparison between both active groups to observe the evolution of cognitive deficits, anxiety-depressive symptoms, and quality of life of the participants just after the application of the interventions and at 6 months of follow-up. Once the analyses are performed, summary tables will be provided for all the planned evaluations: baseline (T0), end of treatment (T1), and follow-up at 6 months (T2). The results will be presented in the form of frequency tables and by means of descriptive statistics (the mean, standard deviation, and percentages) to summarize the characteristics of both the total sample and the participants in each of the three groups. Finally, the results of the study will be reported in accordance with the 2010 statement of the Consolidated Standards of Reporting Trials (CONSORT) [42] and the 2013 guidelines for Standard Protocol Items: Recommendations for Interventional Trials (SPIRIT) [43].

### Interim analyses {21b}

This is not applicable. There are no interim analyses planned.

### Methods for additional analyses (e.g., subgroup analyses) {20b}

This is not applicable. There are no subgroup analyses planned.

### Methods in analysis to handle protocol non-adherence and any statistical methods to handle missing data {20c}

As mentioned above, missing or incomplete data will be considered in the intention-to-treat analysis using the maximum likelihood estimation method.

### Plans to give access to the full protocol, participant-level data and statistical code {31c}

Once the study has been completed and the results published, the corresponding author will provide access to the coded data upon reasonable request.

## Oversight and monitoring

### Composition of the coordinating center and trial steering committee {5d}

The principal investigator and the members of the research team are responsible for monitoring the development of the research.

### Composition of the data monitoring committee, its role, and reporting structure {21a}

Considering the characteristics of this trial (the short duration of the interventions and no expected estimated risks for participants), a data monitoring committee (DMC) will not be necessary

### Adverse event reporting and harms {22}

Patients will be invited to express any discomfort or inconvenience they may experience during the interventions; however, no adverse events are expected to be observed during the development and implementation of the different interventions planned in the trial.

### Frequency and plans for auditing trial conduct {23}

This trial will be monitored by an independent committee of the Andalusian Biomedical Research Ethics Board. This committee requires annual information about the trial for its review, including the number of participants recruited and participants withdrawing. The members of the research group will meet once a week to evaluate the progress of the trial. The interventions of the different groups will be carried out by psychologists with at least a master’s degree in clinical psychology and with prior training in both the UP and the neuropsychological intervention before the start of the interventions. In addition, to address any concerns that may arise during the intervention period, the therapists in charge of carrying out the interventions will be supervised by a coordinator in scheduled coordination meetings.

### Plans for communicating important protocol amendments to relevant parties (e.g. trial participants, ethical committees) {25}

Any possible modification of the protocol during the trial, including changes in study design, sample size, or procedures, will require a modification of the protocol. These possible modifications to the protocol will be agreed upon by the research team, notified to the Andalusian Biomedical Research Ethics Committee and approved prior to their implementation.

### Dissemination plans {31a}

The results of the study will be disseminated at specialized scientific conferences and through publications in peer-reviewed indexed journals in the field of clinical psychology and psycho-oncology. The research team will decide on authorship and order of the authors depending on the contribution of each member.

## Discussion

Cancer patients frequently develop cognitive impairments that negatively affect their quality of life and functional status, especially those who have undergone chemotherapy [3–5]. Several psychological interventions to reduce the frequency of these cognitive alterations have shown positive results. However, a relationship has been observed between emotional well-being (anxiety and depression) and the severity of cognitive alterations in these patients. Therefore, a transdiagnostic intervention can be useful to improve their emotional state and cognitive performance [8–16].

This protocol includes information on a randomized controlled trial in which an intervention based on Barlow’s Unified Protocol (UP) will be carried out with the aim of improving the emotional state and cognitive performance of cancer patients. The UP intervention will be compared to a well-established neuropsychological intervention and a waiting list group. The group assigned to the transdiagnostic intervention group is expected to obtain significantly better outcomes in cognitive performance, anxiety, depression, and quality of life compared to the other two groups.

The planned trial has certain strengths that deserve mention. First, it will include a significant number of participants after estimating the sample size necessary to obtain relevant results. Furthermore, using a randomized controlled trial is an advantage of this protocol, as well as the inclusion of a waitlist control group with which to compare the results. Finally, the intervention based on the transdiagnostic model can help not only to improve the cognitive performance of the participants, but also their emotional state.

Finally, it is necessary to mention some limitations foreseen in this trial. The different treatments which the participants will undergo may influence the results. In addition, differences in the time from completion may influence the participants’ outcomes, although the effect of time on the results is expected to be controlled. Finally, the inclusion of patients with breast cancer only may limit the usefulness of the results, since cognitive alterations appear in cancer patients in general.

Despite these limitations, the expected results of this randomized and controlled trial can help to achieve a comprehensive improvement in cancer patients in relevant areas such as cognitive function and emotional state.

## Trial status

Protocol version v01. 12 March 2022. This trial is currently ongoing, with activity and recruitment beginning on April 25, 2022. Recruitment completion is expected on August 31, 2022

## Supplementary Information

**Additional file 1.**

**Additional file 2.**

## Data Availability

Only the members of the research team will have access to study data.

## References

[1] World Health Organization (WHO) (2022). Breast cancer.

[2] REDECAN. Red Española de Registros de Cáncer. Retrieved 17 Jan 2022 from: https://www.redecan.org

[3] Hutterer M, Oberndorfer S (2021). Cognitive impairment in cancer patients and survivors—clinical presentation, pathophysiology, diagnosis and management. memo-Magazine of European. Med Oncol.

[4] Jia M, Zhang X, Wei L, Gao J (2021). Measurement, outcomes and interventions of cognitive function after breast cancer treatment: a narrative review. Asia Pac J Clin Oncol.

[5] Shaw C, Baldwin A, Anderson C (2021). Cognitive effects of chemotherapy: An integrative review. Eur J Oncol Nurs.

[6] Wefel JS, Saleeba AK, Buzdar AU, Meyers CA (2010). Acute and late onset cognitive dysfunction associated with chemotherapy in women with breast cancer. Cancer.

[7] Collins B, MacKenzie J, Tasca GA, Scherling C, Smith A (2014). Persistent cognitive changes in breast cancer patients 1 year following completion of chemotherapy. J Int Neuropsychol Soc.

[8] King S, Green HJ (2015). Psychological intervention for improving cognitive function in cancer survivors: a literature review and randomized controlled trial. Front Oncol.

[9] Ercoli LM, Petersen L, Hunter AM, Castellon SA, Kwan L, Kahn-Mills BA (2015). Cognitive rehabilitation group intervention for breast cancer survivors: results of a randomized clinical trial. Psychooncology.

[10] Bray VJ, Dhillon HM, Bell ML, Kabourakis M, Fiero MH, Yip D (2017). Evaluation of a web-based cognitive rehabilitation program in cancer survivors reporting cognitive symptoms after chemotherapy. Am Soc Clin Oncol.

[11] Bail J, Meneses K (2016). Computer-Based Cognitive Training for Chemotherapy-Related Cognitive Impairment in Breast Cancer Survivors. Clin J Oncol Nurs.

[12] Dos Santos M, Hardy-Léger I, Rigal O, Licaj I, Dauchy S, Levy C (2020). Cognitive rehabilitation program to improve cognition of cancer patients treated with chemotherapy: A 3-arm randomized trial. Cancer.

[13] Atallah M, Cooper B, Muñoz RF, Paul SM, Anguera J, Levine JD (2020). Psychological symptoms and stress are associated with decrements in attentional function in cancer patients undergoing chemotherapy. Cancer Nurs.

[14] Henneghan A, Stuifbergen A, Becker H, Kesler S, King E (2018). Modifiable correlates of perceived cognitive function in breast cancer survivors up to 10 years after chemotherapy completion. J Cancer Surviv.

[15] Menning S, de Ruiter MB, Kieffer JM, van Rentergem JA, Veltman DJ, Fruijtier A (2016). Cognitive impairment in a subset of breast cancer patients after systemic therapy—results from a longitudinal study. J Pain Symptom Manag.

[16] Ramalho M, Fontes F, Ruano L, Pereira S, Lunet N (2017). Cognitive impairment in the first year after breast cancer diagnosis: a prospective cohort study. Breast.

[17] Von Ah D, Carpenter JS, Saykin A, Monahan P, Wu J, Yu M (2012). Advanced cognitive training for breast cancer survivors: A randomized controlled trial. Breast Cancer Res Treat.

[18] Jobe JB, Smith DM, Ball K, Tennstedt SL, Marsiske M, Willis SL, Rebok GW, Morris JN, Helmers KF, Leveck MD, Kleinman K (2001). ACTIVE: a cognitive intervention trial to promote independence in older adults. Control Clin Trials.

[19] Mahncke HW, Connor BB, Appelman J, Ahsanuddin ON, Hardy JL, Wood RA, Joyce NM, Boniske T, Atkins SM, Merzenich MM (2006). Memory enhancement in healthy older adults using a brain plasticity-based training program: a randomized, controlled study. Proc Natl Acad Sci U S A.

[20] Barlow DH, Farchione TJ, Fairholme CP, Ellard KK, Boisseau CL, Allen LB, Ehrenreich-May J (2011). The unified protocol for transdiagnostic treatment of emotional disorders: Therapist guide.

[21] Barlow DH, Farchione TJ, Fairholme CP, Ellard KK, Boisseau CL, Allen LB, Ehrenreich-May J (2015). Protocolo unificado para el tratamiento transdiagnóstico de los trastornos emocionales: Manual del terapeuta y manual del paciente [The unified protocol for transdiagnostic treatment of emotional disorders: Client workbook and Therapist guide].

[22] Ono M, Ogilvie JM, Wilson JS, Green HJ, Chambers SK, Ownsworth T, Shum DHK (2015). A Meta-Analysis of Cognitive Impairment and Decline Associated with Adjuvant Chemotherapy in Women with Breast Cancer. Front Oncol.

[23] Hermelink K, Untch M, Lux MP, Kreienberg R, Beck T, Bauerfeind I, Münzel K (2007). Cognitive function during neoadjuvant chemotherapy for breast cancer: results of a prospective, multicenter, longitudinal study. Cancer.

[24] Zwerenz R, Beutel ME, Imruck BH, Wiltink J, Haselbacher A, Ruckes C, Schmidberger H, Hoffmann G, Schmidt M, Köhler U, Langanke D, Kortmann RD, Kuhnt S, Weissflog G, Barthel Y, Leuteritz K (2012). Efficacy of psychodynamic short-term psychotherapy for depressed breast cancer patients: study protocol for a randomized controlled trial. BMC Cancer.

[25] Osoba D, Rodrigues G, Myles J, Zee B, Pater J (1998). Interpreting the significance of changes in health-related quality-of-life scores. J Clin Oncol.

[26] Snyder CF, Blackford AL, Sussman J, Bainbridge D, Howell D, Seow HY (2015). Identifying changes in scores on the EORTC-QLQ-C30 representing a change in patients’ supportive care needs. Qual Life Res.

[27] Folstein MF, Folstein SE, McHugh PR (1975). Mini-mental state: A practical method for grading the cognitive state of patients for the clinician. J Psychiatr Res.

[28] Blesa R, Pujol M, Aguilar M, Santacruz P, Bertran-Serra I, Hernández G, Crespo MC (2001). Clinical validity of the “mini-mental state” for Spanish speaking communities. Neuropsychologia.

[29] Park JH, Bae SH, Jung YS, Jung YM (2015). The psychometric properties of the Korean version of the functional assessment of cancer therapy-cognitive (FACT-Cog) in Korean patients with breast cancer. Support Care Cancer.

[30] Joly F, Lange M, Rigal O, Correia H, Giffard B, Beaumont JL (2012). French version of the functional assessment of cancer therapy–cognitive function (FACT-Cog) version 3. Support Care Cancer.

[31] Lozoya-Delgado P, de León JMRS, Pedrero-Pérez EJ (2012). Validación de un cuestionario de quejas cognitivas para adultos jóvenes: Relación entre las quejas subjetivas de memoria, la sintomatología prefrontal y el estrés percibido. Rev Neurol.

[32] Wefel JS, Vardy J, Ahles T, Schagen SB (2011). International Cognition and Cancer Task Force recommendations to harmonise studies of cognitive function in patients with cancer. Lancet Oncol.

[33] Brandt J, Benedict HRB (2001). Hopkins Verbal Learning Test-revised professional manual.

[34] Reitan RM (1958). Validity of the trail making test as an indicator of organic brain damage. Percept Motor Skills.

[35] Benton A, Sd H, Sivan A (1983). Multilingual aplasia examination.

[36] Spreen O, Benton AL (1969). Neurosensory Center Comprehensive Examination for Aphasia.

[37] Zigmond A, Snaith R (1983). The Hospital Anxiety and Depression Scale. Acta Psychiatr Scand.

[38] López-Roig S, Terol MC, Pastor MA, Massutí B, Rodríguez-Marín J, Neipp MC, Leyda JI, Martín-Aragón M, Sánchez S, y Sitges, E. (2000). Ansiedad y Depresión. Validación de la escala HAD en pacientes oncológicos. Rev Psicol Salud.

[39] Aaronson NK, Ahmedzai S, Bergman B, Bullinger M, Cull A, Duez NJ (1993). The European Organization for Research and Treatment of Cancer QLQ-C30: a quality-of-life instrument for use in international clinical trials in oncology. J Natl Cancer Inst.

[40] Sprangers MA, Groenvold M, Arraras JI, Franklin J, te Velde A, Muller M (1996). The European Organization for Research and Treatment of Cancer breast cancer-specific quality-of-life questionnaire module: first results from a three-country field study. J Clin Oncol.

[41] Gueorguieva R, Krystal JH (2004). Move over ANOVA: progress in analyzing repeated-measures data and its reflection in paper published in the Archives of General Psychiatry. Arch Gen Psychiatry.

[42] Schulz KF, Altman DG, Moher D, Consort Group (2010). CONSORT 2010 statement: updated guidelines for reporting parallel group randomised trials. BMJ.

[43] Moher D, Hopewell S, Schulz KF, Montori V, Gøtzsche PC, Devereaux PJ, Altman DG (2010). CONSORT 2010 explanation and elaboration: updated guidelines for reporting parallel group randomised trials. BMJ.

