# Supplementary material for: Effectiveness of the Unified Barlow Protocol (UP) and neuropsychological treatment in cancer survivors for cognitive impairments: study protocol for a randomized controlled trial
Source: Trials. 2022 Sep 30;23:819. doi: 10.1186/s13063-022-06731-w (PMC9524048; doi:10.1186/s13063-022-06731-w)
Supplement: Supplementary file 1 — Additional file 1. [file 13063_2022_6731_MOESM1_ESM.docx]

| Table 1. WHO data set for clinical trials | |
| --- | --- |
| ****Data category**** | Information |
| **Primary Registry and Trial Identifying Number** | ClinicalTrials.gov Identifier: NCT05289258 |
| **Date of Registration in Primary Registry** | March 21, 2022 |
| **Secondary Identifying Numbers** |  |
| **Source(s) of Monetary or Material Support** | European Regional Development Fund (ERDF) , grant number: 1380800-R |
| **Primary Sponsor** | University of Cordoba |
| **Secondary Sponsor(s)** |  |
| **Contact for Public Queries** | FGT, [z12gatof@uco.es](mailto:z12gatof@uco.es) |
| **Contact for Scientific Queries** | FGT, [z12gatof@uco.es](mailto:z12gatof@uco.es) |
| **Public Title** | Unified Barlow Protocol (UP) in Cancer Survivors for Cognitive Impairments |
| **Scientific Title** | Effectiveness of the Unified Barlow Protocol (UP) and neuropsychological treatment in cancer survivors for cognitive impairments: study protocol for a randomized controlled trial |
| **Countries of Recruitment** | Spain |
| **Health Condition(s) or Problem(s) Studied** | Cancer |
| **Intervention(s)** | - Unified Protocol for the Transdiagnostic Treatment of Emotional Disorders (PU). - Neuropsychological treatment - Waitlist group |
| **Key Inclusion and Exclusion Criteria** | Inclusion criteria: Cancer diagnosis, stages I-III / Cancer type: Breast / Have received the last chemotherapy session in the last 6 months and a maximum of 6 years of treatment completion / Probable or mild to moderate cognitive impairment (score between 26 and 10 points according to the MMSE) / Ability to be fluent in Spanish / Not currently participating in another clinical trial. Not currently receiving other psychological treatment  Exclusion Criteria: Men and women aged > 70 years / Diagnosis of cancer, stage IV or other types of cancer / Last chemotherapy session < 6 months or > 6 years / No cognitive impairment (MMSE score between 30 and 27 points) / Diagnosis of mental disorder (including substance abuse) prior to cancer diagnosis / Relapse in disease after chemotherapy treatment is completed / Neurodevelopmental Disorder Diagnosis / Diagnosis of diseases that affect cognitive performance such as: hypertension, cardiac diseases, epilepsy, dementias, multiple sclerosis, functional disorders (fibromyalgia, chronic fatigue syndrome, irritable bowel syndrome, post-concussion syndrome, whiplash syndrome), CNS infections (HIV, encephalitis), metabolic disorders (diabetes, B12 deficiency), obstructive sleep apnea, brain damage (stroke, TBI, CNS cancer) and use of medications / substances that interfere with cognitive function such as pregabalin, gabapentin, topiramate, antidepressants tricyclics, sodium valproate, anticholinergics, methylphenidate, typical antipsychotics. |
| **Study Type** | This study is a three-arm controlled, randomised superiority trial, with a pre-post-follow-up measures intergroup design with a 1:1:1 allocation ratio |
| **Date of First Enrollment** | In process |
| **Sample Size** | 123 total participants (41 patients for each study arm) |
| **Recruitment Status** | Recruiting |
| **Primary Outcome(s)** | Outcome Name: Psychological flexibility Metric/method of measurement: AAQ-II questionnaire Timepoint: post intervention, 3 and 6 months after intervention |
| **Key Secondary Outcomes** | Outcome Name: Anxiety and Depression Metric/method of measurement: Hospital Anxiety and Depression Scale (HADS) Timepoint: post intervention, 3 and 6 months after intervention  Outcome Name: Fatigue Metric/method of measurement: Brief Fatigue Inventory (BFI) Timepoint: post intervention, 3 and 6 months after intervention  Outcome Name: Insomnia Metric/method of measurement: Insomnia Severity Index (ISI). Timepoint: post intervention, 3 and 6 months after intervention  Outcome Name: Post-traumatic Growth Metric/method of measurement: Post-traumatic Growth Inventory-short version (PTGI-SF) Timepoint: post intervention, 3 and 6 months after intervention  Outcome Name: Quality of Life Metric/method of measurement: EORTC QLQ C-30 (version 3) Timepoint: post intervention, 3 and 6 months after intervention |
| **Ethics Review** | This research obtained the approval from the Andalusian Biomedical Research Ethics (ref. number: 5292) |
| **Completion date** | 31th December 2022 |
| Summary Results | No data are currently collected. |
| **IPD sharing statement** | Plan to share IPD: No |
