# Supplementary material for: Effectiveness of the Unified Barlow Protocol (UP) and neuropsychological treatment in cancer survivors for cognitive impairments: study protocol for a randomized controlled trial
Source: Trials. 2022 Sep 30;23:819. doi: 10.1186/s13063-022-06731-w (PMC9524048; doi:10.1186/s13063-022-06731-w)
Supplement: Supplementary file 2 — Additional file 2. [file 13063_2022_6731_MOESM2_ESM.doc]

**CONSENTIMIENTO INFORMADO – CONSENTIMIENTO POR ESCRITO DEL PACIENTE**

Yo, (Nombre y Apellidos):................................................................................................................................

- He leído el documento informativo que acompaña a este consentimiento (Información al Paciente).
- He podido hacer preguntas sobre el estudio: **Efectividad de la rehabilitación cognitiva y el protocolo unificado de Barlow (UP) en supervivientes al cáncer: un ensayo aleatorizado y controlado.**
- He recibido suficiente información sobre el estudio: **Efectividad de la rehabilitación cognitiva y el protocolo unificado de Barlow (UP) en supervivientes al cáncer: un ensayo aleatorizado y controlado.**
- He hablado con el profesional sanitario informador: …………………………………………………………

- Comprendo que mi participación es voluntaria y soy libre de participar o no en el estudio.
- Se me ha informado que todos los datos obtenidos en este estudio serán confidenciales y se tratarán conforme establece la [Ley Orgánica 3/2018 del 6 de Diciembre de 2018 de Protección de Datos Personales y garantía de los derechos digitales](https://es.wikipedia.org/wiki/Ley_Orgánica_de_Protección_de_Datos_Personales_y_garantía_de_los_derechos_digitales),
- Se me ha informado de que la información obtenida sólo se utilizará para los fines específicos del estudio.
- La participación en el presente estudio no conlleva ningún riesgo para los participantes, que podrán abandonar el estudio en cualquier momento.
- **Deseo** ser informado/a de mis datos de carácter personal que se obtengan en el curso de la investigación, incluidos los descubrimientos inesperados que se puedan producir, siempre que esta información sea necesaria para evitar un grave perjuicio para mi salud o la de mis familiares biológicos: **Si No**

Comprendo que puedo retirarme del estudio:

- Cuando quiera
- Sin tener que dar explicaciones
- Sin que esto repercuta en mis cuidados médicos
- Presto libremente mi conformidad para participar en el proyecto titulado: **Efectividad de la rehabilitación cognitiva y el protocolo unificado de Barlow (UP) en supervivientes al cáncer: un ensayo aleatorizado y controlado.**

Firma del paciente Firma del profesional

(o representante legal en su caso) sanitario informador

Nombre y apellidos:……………………………………. Nombre y apellidos: ……………………………………

Fecha: …………………………………………………. Fecha: …………………………………………………...

**REVOCACIÓN CONSENTIMIENTO INFORMADO**

- Yo, (Nombre y Apellidos):................................................................................................................................, de forma libre y consciente he decidido retirar el consentimiento para participar en el proyecto titulado: **Efectividad de la rehabilitación cognitiva y el protocolo unificado de Barlow (UP) en supervivientes al cáncer: un ensayo aleatorizado y controlado.** sin que eso me cause perjuicio alguno tal y como se especifica en el presente documento.

Firma del paciente Firma del profesional

(o representante legal en su caso) sanitario informador

Nombre y apellidos:……………………… Nombre y apellidos: …………………………………

Fecha: ……………………………………. Fecha: ………………………………………………..
